# Quantitative comparison between carotid plaque hardness and histopathological findings: an observational study

**DOI:** 10.1186/s13000-022-01239-y

**Published:** 2022-07-11

**Authors:** Daisuke Fukushima, Kosuke Kondo, Naoyuki Harada, Sayaka Terazono, Kei Uchino, Kazutoshi Shibuya, Nobuo Sugo

**Affiliations:** 1 Department of Neurosurgery (Omori), School of Medicine, Faculty of Medicine, Toho University, Tokyo, 143-8541 Japan; 2 Department of Pathology, Toho University Omori Medical Center, Tokyo, Japan

**Keywords:** Carotid artery stenosis, Plaque hardness, Carotid endarterectomy, Carotid artery stenting, Collagen, Calcification

## Abstract

**Background:**

Plaque hardness in carotid artery stenosis correlates with cerebral infarction. This study aimed to quantitatively compare plaque hardness with histopathological findings and identify the pathological factors involved in plaque hardness.

**Methods:**

This study included 84 patients (89 lesions) undergoing carotid endarterectomy (CEA) at our institution. Plaque hardness was quantitatively measured immediately after excision using a hardness meter. Collagen and calcification were evaluated as the pathological factors. Collagen was stained with Elastica van Gieson stain, converted to a gray-scale image, and displayed in a 256-step histogram. The median gray-scale median (GSM) was used as the collagen content. The degree of calcification was defined by the hematoxylin–eosin stain as follows: "0:" no calcification, "1:" scattered microcalcification, or "2:" calcification greater than 1 mm or more than 2% of the total calcification. Carotid echocardiographic findings, specifically echoluminance or the brightness of the narrowest lesion of the plaque, classified as hypo-, iso-, or hyper-echoic by comparison with the intima-media complex surrounding the plaque, and clinical data were reviewed.

**Results:**

Plaque hardness was significantly negatively correlated with GSM [Spearman's correlation coefficient: -0.7137 (*p* < 0.0001)]: the harder the plaque, the higher the collagen content. There were significant differences between plaque hardness and degree of calcification between "0" and "2" (*p* = 0.0206). For plaque hardness and echoluminance (hypo-iso-hyper), significant differences were found between hypo-iso (*p* = 0.0220), hypo-hyper (*p* = 0.0006), and iso-hyper (*p* = 0.0015): the harder the plaque, the higher the luminance. In single regression analysis, GSM, sex, and diabetes mellitus were significant variables, and in multiple regression analysis, only GSM was extracted as a significant variable.

**Conclusions:**

Plaque hardness was associated with a higher amount of collagen, which is the main component of the fibrous cap. Greater plaque hardness was associated with increased plaque stability. The degree of calcification may also be associated with plaque hardness.

## Background

Carotid artery stenosis is one of the most common atherosclerotic diseases. The frequency of asymptomatic carotid artery stenosis in the general public is 0–7.5% for moderate stenosis and 0–3.1% for severe stenosis [1]. Carotid artery stenosis is caused by hypertension, diabetes, dyslipidemia, and smoking, and often occurs at the carotid bifurcation; it is defined as a plaque when its localized intimal thickening is greater than 1.1 mm [2].

The main mechanism of cerebral infarction caused by carotid artery stenosis is the reduction of cerebral blood flow due to stenosis associated with thickening of the plaque [3–5] and scattering of debris when fragile and soft plaques rupture [6–8]. Stenosis detected by cerebral angiography and carotid echocardiography are important indications for surgery for carotid artery stenosis, according to the Japanese Guidelines for the Management of Stroke 2021. However, indications for surgery are not based on plaque properties. Therefore, plaque hardness may be an important finding that strongly influences the pathogenesis, therapeutic indications, and postoperative complications of carotid artery stenosis.

Carotid endarterectomy (CEA). and carotid artery stenting (CAS) are typical surgical procedures for carotid artery stenosis. CEA involves surgically removing the plaque from the entire vascular intima [9], while CAS is an interventional therapy in which a metal stent is placed in the stenotic carotid artery to dilate it [10, 11].

In the case of carotid artery stenosis with soft unstable plaques, the risk of postoperative cerebral infarction is increased because the debris is easily dispersed by physical traction and stimulation during CEA surgery [12–14]. Conversely, if the plaque is hard, the tunica media may also be removed alongside the intima when the plaque is peeled off the vessel wall. Consequently, there is a risk of vessel thinning and perforation. In other words, preoperative plaque hardness provides useful information in preoperative and perioperative simulations because the precautions taken during surgical manipulation in CEA differ depending on plaque hardness.

It has been reported that during CAS, catheter manipulation can disrupt the fibrous cap causing the soft unstable plaque to disperse and embolize [15, 16]. Additionally, with hard plaques, CAS can cause plaque divergence, bradycardia due to overdilation, and restenosis due to inadequate dilation [17]. Hence, when performing CAS as well as CEA, preoperative plaque hardness is also an important finding, particularly in the determination of the type of surgery required and the device to be used, as it affects postoperative outcomes.

Several studies on plaque hardness have been reported, although most were based on subjective evaluation of hardness by the surgeon [7, 8, 18]. Although some studies have assessed plaque hardness, few have assessed quantitative measurements of the plaque, and have multiple limitations. Marher et al. performed quantitative measurements by tensile and compressive tests in 14 fresh plaques removed by CEA and compared these with echogenic findings. The results showed that calcified plaques were stiffer and hypoechoic plaques were softer in the compressive test [19]. Antonacci et al. quantitatively measured plaque hardness in mice using Brillouin microscopy, a non-contact indirect measurement. Pathological results suggested that plaque hardness was correlated with collagen abundance and inversely correlated with fat content [20]. In our previous research, we measured the hardness of various general substances, such as tofu, sliced cheese, a plastic eraser, and a stick of gum, with a hardness meter and proved the reproducibility and accuracy of the device. We also compared plaque hardness with echocardiographic findings in 44 cases, and the results showed that the harder the plaque, the higher the echo luminance [21].

Moreover, the cause and pathological basis for different plaque hardness in each case remain unknown. This study aimed to quantitatively compare plaque hardness with histopathological findings and to identify the pathological factors involved in plaque hardness.

## Methods

### Patient population

Eighty-nine lesions in 84 patients who underwent CEA at our institution between December 2009 and May 2018 were included in this study.

### Surgical procedure

CEA in all cases was performed with the aid of a microscope, with the patient under general anesthesia. An incision was made along the anterior border of the sternocleidomastoid muscle, and the platysma was cut. The sternocleidomastoid muscle was dissected to expose the common, internal, and external carotid arteries, and superior thyroid artery. Each vessel was occluded with forceps, and a longitudinal incision was made from the common carotid artery to the internal carotid artery. After inserting a carotid shunt, the plaque was removed as a single mass along with the entire endothelium.

### Quantitative measurement with hardness meter

**Fig. 1:**
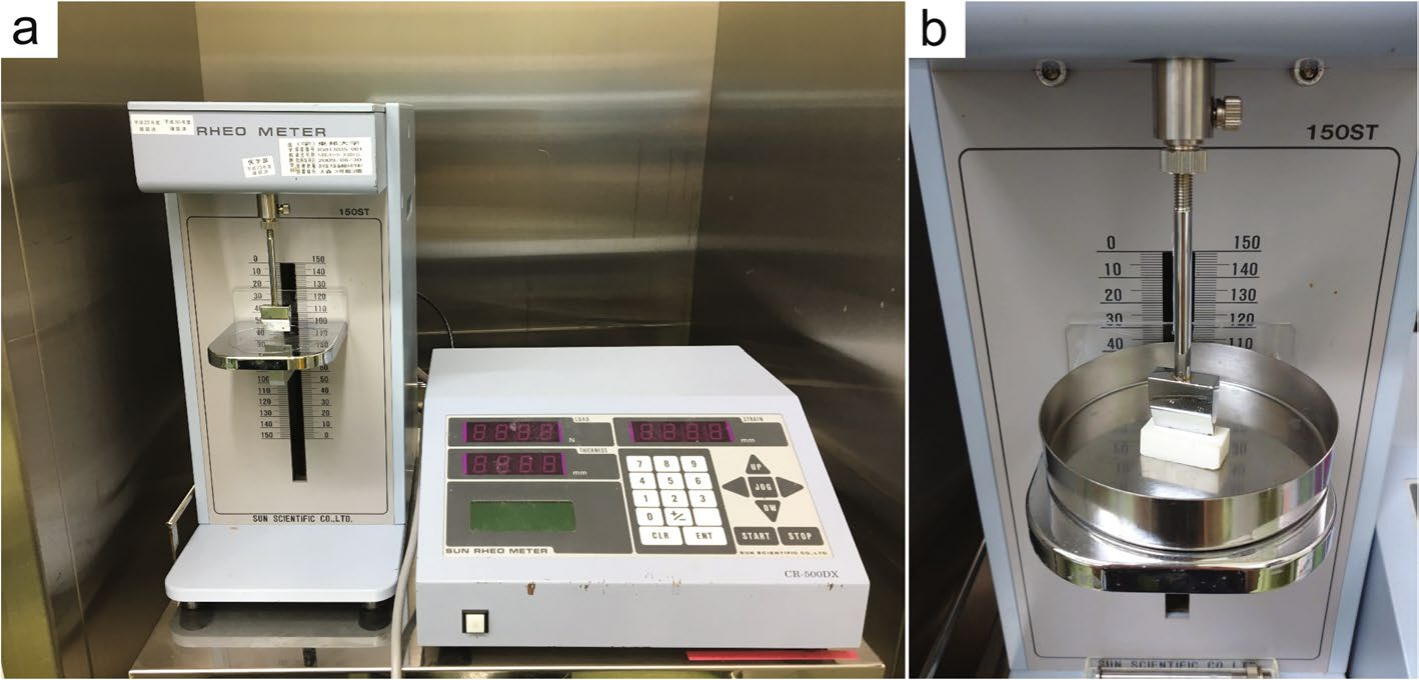
**a** Hardness meter with special adaptor used to obtain the measurements. The plaque was placed on the measurement table and measured by applying pressure using a special adaptor. **b** The special adaptor compresses and measures the sample

A hardness meter (Rheometer CR-500DX-SII: SUN SCIENTIFIC CO., LTD, Tokyo, Japan) was installed in the operating room (Fig. 1a). This instrument is a commercially available industrial product for measuring the hardness of foods, resins, and pharmaceuticals. It measures the viscosity of a liquid and the elasticity of a solid. A sample was placed on the measurement table, and the upper special adaptor was moved to apply a small compressive pre- load of 0.1 N to the sample at a cross head speed of 0.1 mm/s (Fig. 1b).

The analysis software used was RheoData Analyzer VR.2.8g3 (SUN SCIENTIFIC CO. LTD, Tokyo, Japan). The unit of hardness was expressed in megapascals (MPa). Measurements were obtained within 1 h of removal.

### Histopathological evaluation

**Fig. 2:**
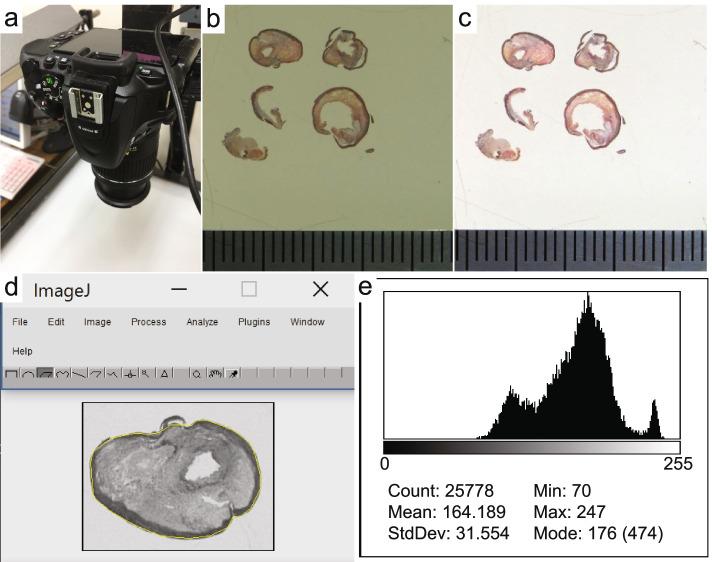
**a** Single-lens reflex digital camera fixed on the table for specimen photography. **b** Image of EVG-stained specimen taken by the camera. **c** Post-automatic image quality adjustment. **d** Grayscale converted image manually surrounded by ROIs of intima and plaque. **e** Grayscale image converted to a histogram and the median value determined

Collagen is a major constituent of normal blood vessel walls; it is also present in the plaque and has high rigidity [22, 23]. Therefore, we focused on collagen as one of the pathological factors related to plaque hardness and performed Elastica van Gieson (EVG) staining of collagen and elastin. Calcification was also assessed using hematoxylin–eosin staining (HE). The resected plaques were fixed with 10% formalin immediately after hardness measurement in the operating room. Image analysis and quantitative evaluation of EVG- and HE-stained specimens were performed using the following methods:

Plaque tissues were fixed for 24 to 48 h, paraffin-embedded blocks were prepared and 3-μm-thick sections were mounted on glass slides and stained with HE and EVG stains. The EVG-stained specimens were placed on a table for photography using a fixed digital camera (Nikon SLR; Nikon Corporation, Tokyo, Japan) (Fig. 2a and b). In order to eliminate errors in the image of each sample taken with a digital camera, automatic image adjustment was performed on all samples and then converted to grayscale images using image editing software (Adobe Photoshop Elements 2018, Adobe Inc. California, USA) (Fig. 2 c and d). Auto-contrast does not adjust each color channel individually, so it does not remove or create unwanted tints. Then, using image analysis software [Image J: Wayne Rasband (NIH), MD, USA], bounding rectangles were manually set up with four adjacent points, and plot profiles were constructed. Specifically, the region of interest of the image converted to grayscale was chosen using polygon selection (Fig. 2). The image was then converted for analysis to a histogram. The grayscale images of the intima and plaque were converted to a 256-step histogram from 0 to 255 and displayed. The median value of the gray-scale median (GSM) was evaluated as the collagen content (Fig. 2 e). In this grayscale image, the darkest value of 0 indicates the highest collagen content, and the lightest value of 255 indicates the lowest collagen content.

On HE-stained specimens, the degree of calcification was defined as follows: "0" was no calcification, "1" was scattered microcalcification, and "2" was calcification of 1 mm or more or microcalcification of 2% or more of the total.

### Clinical and preoperative imaging findings

Clinical findings included age, sex, symptomatic or asymptomatic, medical history (hypertension, lipid metabolism disorders, diabetes mellitus), smoking status, and blood test results.

The preoperative stenosis rate for carotid artery stenosis was measured using the North American Symptomatic Carotid Endarterectomy Trial method on digital subtraction angiography (AREX-VC830A; Canon Medical Systems Corp., Tochigi, Japan) [24].

The XARIO SSA-660A system (Canon Medical Systems Corp., Tochigi, Japan) was used for carotid artery echocardiography, and PLT-805AT (Canon Medical Systems Corp., Tochigi, Japan) and PLT-704AT (Canon Medical Systems Corp., Tochigi, Japan) were used as transducers. Echoluminance is the brightness of the narrowest lesion of the plaque and was classified as hypo-, iso-, or hyper-echoic by comparison with the intima-media complex surrounding the plaque. Postoperative ischemic complications were assessed using diffusion-weighted images on MRI (1.5 T Magnetom Avanto, Siemens Healthcare, Erlangen, Germany) performed within 1 week after surgery.

### Statistical analysis

Continuous variables are reported as mean ± standard deviation, and categorical variables are reported as counts and percentages. For comparisons between two groups, the chi-squared or Fisher's exact test was used for categorical variables, and the Student's t-test was used for categorical variables, as appropriate. Multiple comparisons using the Kruskal–Wallis test and the Steel–Dwass method were made between the three groups. Statistical significance was set at *p* < 0.05. Individual correlations between plaque hardness, pathological findings, and clinical data were examined using single regression analysis. In multiple regression analysis, all variables were first entered and then selected using the stepwise variable reduction method. The results are reported as partial regression coefficients, 95% confidence intervals, and p-values. IBM SPSS Statistics 26 (IBM Corp., Armonk, NY, USA) was used for data analysis.

This study was approved by the Ethics Committee of the School of Medicine, Faculty of Medicine, Toho University (approval number: A16026-25006) and the Ethics Committee of Toho University Omori Medical Center (approval number: M20250).

## Results

Patient background, clinical data, and imaging findings for the 84 patients included in this study are summarized in Table 1. There were 76 men (90.5%), with a mean age of 70.6 ± 7.1 years [median 42–82], 37 symptomatic cases (42.6%), and 35 (39.3%) cases of diabetes mellitus. Echocardiographic findings were hypoechoic in 7 cases (7.9%), isoechoic in 42 cases (47.2%), and hyperechoic in 36 cases (40.4%).

**Table 1:** Summary of cases

No. of patient	84
No. of lesion	89
Sex [M:F]	76:8
Age	70.6 ± 7.1 (42–82)
Symptoms	37[42.6%]
HT	71[79.8%]
DL	41[46.1%]
DM	35[39.3%]
T-cho	173.8 ± 37.9 (102–294)
HbA1c	6.1 ± 0.7 (4.7–9.2)
stenosis rate	69.5 ± 17.9 (12–100)
Echo finding	
Low	7
Iso	42
High	36
Smoking status	68[76.4%]
DWI high	4 [4.5%]

*HT* Hypertension, *DL* Dyslipidemia, *DM* Diabetes mellitus, *T-cho* Total cholesterol, *DWI* Diffusion-weighted imaging

The quantitatively measured plaque hardness and GSM obtained from EVG staining, and the degree of calcification on HE staining are also shown in Table 2. The mean stiffness value and GSM for all plaques was 4.0 ± 4.4 MPa and 175.7 ± 16.7 (mean ± standard deviation), respectively, and calcification was “0” in 28 cases (31.5%), “1” in 38 cases (42.7%), and “2” in 23 cases (25.8%).

**Table 2:** Plaque hardness and GSM, degree of calcification

Plaque Hardness (MPa)	4.0 ± 4.4 (0.13–31.8)
Gray scale median	175.7 ± 16.7 (131.7–219.2)
Calcification	
0	28
1	38
2	23

"0" was no calcification, "1" was scattered microcalcification, and "2" was calcification of 1 mm or more or the presence of microcalcification of 2% or more of the total

**Fig. 3:**
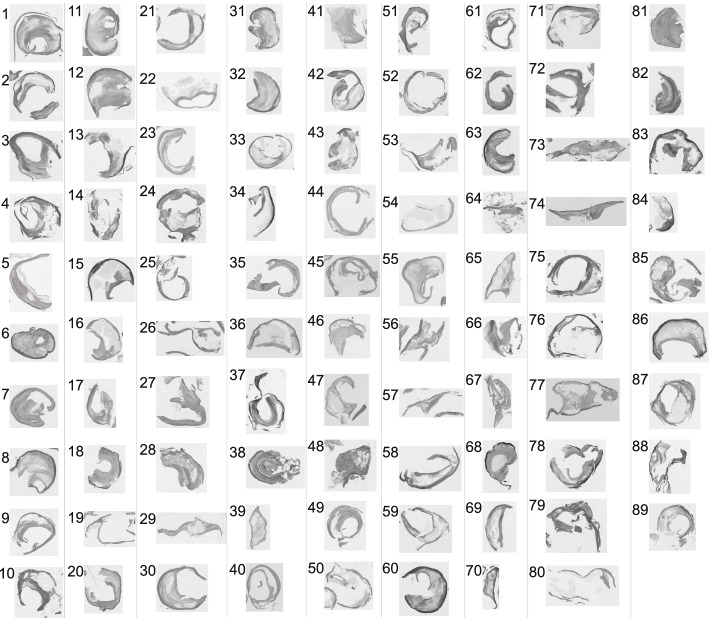
Grayscale images of all cases

Grayscale images of all plaques in this study are presented in Fig. 3.

**Fig. 4:**
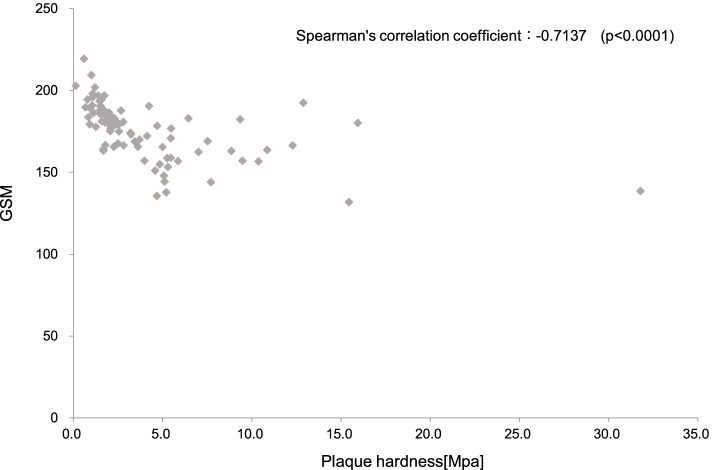
Plaque hardness was negatively correlated with GSM from EVG [Spearman's correlation coefficient: -0.7137 (*p* < 0.0001)], indicating that hard plaques are rich in collagen

Plaque hardness was significantly negatively correlated with GSM from EVG [Spearman's correlation coefficient: -0.7137 (*p* < 0.0001)], indicating that hard plaques are rich in collagen (Fig. 4).

**Fig. 5:**
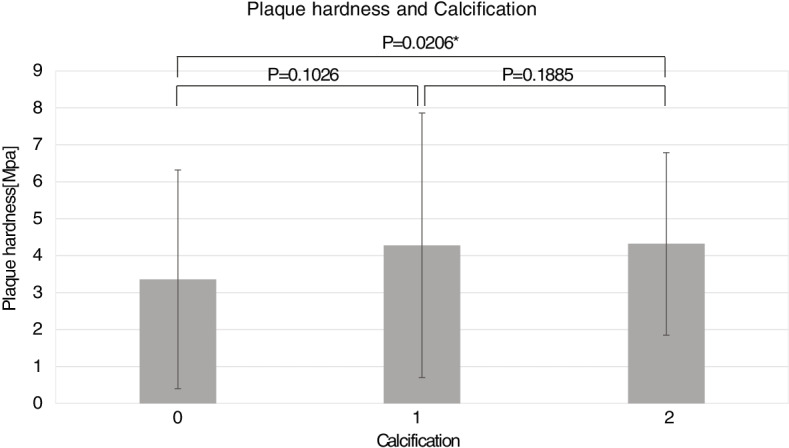
Comparison of plaque hardness and semi-quantified calcification. "0" was no calcification, "1" was scattered microcalcification, and "2" was calcification of 1 mm or more or the presence of microcalcification of 2% or more of the total

The relationship between plaque hardness and the degree of calcification in HE showed a significant difference between "0" and "2" (*p* = 0.0206) (Fig. 5), and plaque containing a large amount of calcification was harder than the uncalcified plaque.

**Fig. 6:**
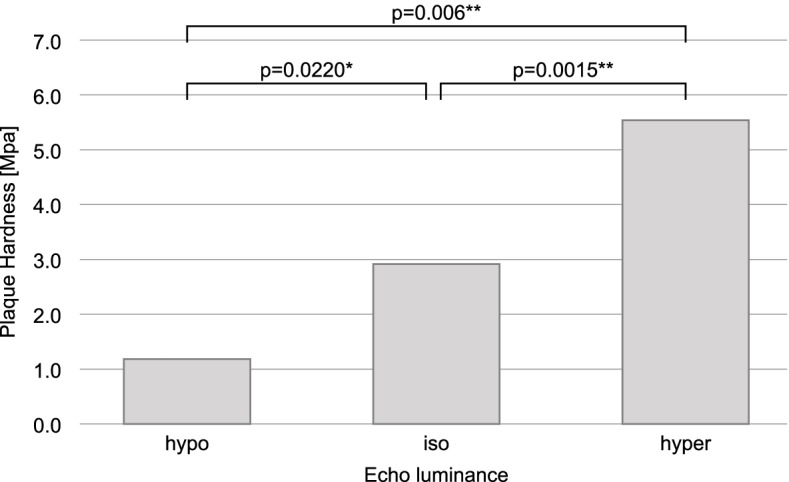
Comparison of plaque hardness and echo luminance (hypo, iso, hyper-echoic). The brightness of the narrowest region of the plaque was classified as hypo-echoic, isoechoic, or hyper-echoic based on the brightness of the intima-media complex near the plaque

In multiple comparisons of plaque hardness and echoluminance (low-iso-high), statistically significant differences were observed in low-iso (*p* = 0.0220), low–high (*p* = 0.0006), and iso-high (*p* = 0.0015); that is, harder plaques were associated with higher luminance (Fig. 6).

**Fig. 7:**
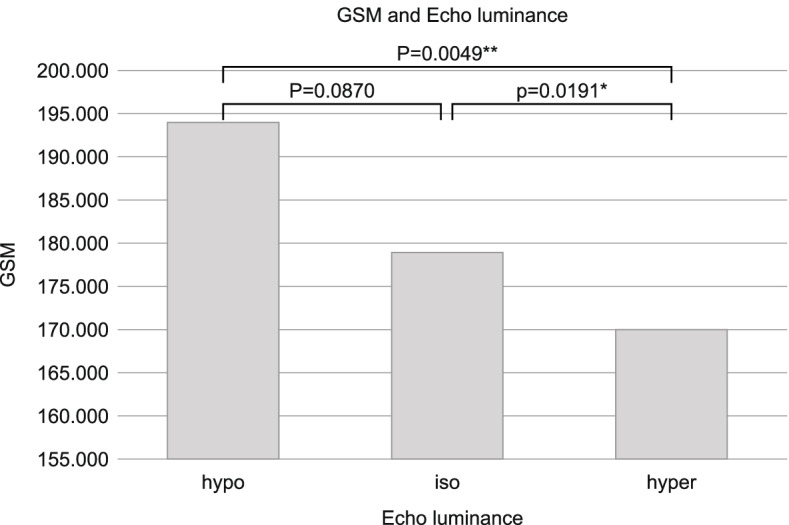
Comparison of GSM and echo brightness (hypo, iso, hyper-echoic). The brightness of the narrowest region of the plaque was classified as hypo-echoic, iso-echoic, or hyper-echoic based on the brightness of the intima-media complex near the plaque

Multiple comparisons of GSM and echoluminance showed differences in low–high (*p* = 0.0049) and iso-high (*p* = 0.0191) echoluminance, with high echoluminance showing low GSM reflecting abundant collagen (Fig. 7).

GSM, calcification, sex, age, symptoms, hypertension, dyslipidemia, diabetes mellitus, smoking status, total cholesterol, HbA1c, angiographic stenosis rate, and plaque hardness were used as objective variables to examine factors that correlate with plaque hardness and as explanatory variables for regression analysis. First, in the simple regression analysis, GSM, sex, and diabetes mellitus were significant (Table 3). In the multiple regression analysis, only GSM was extracted as a significant variable (Table 4).

**Table 3:** Single regression analysis of plaque hardness and patient background

Variable	partial regression coefficient	standard error	95% confidence interval for partial regression coefficient		*P* value
			lower limit	upper limit	
GSM	-0.1412	0.0238	-0.1885	-0.0938	*p* < 0.0001**
Degree of pathological calcification(0–2)	0.4985	0.6235	-0.7408	1.7378	0.4262
Sex(M0F1)	4.1369	1.5910	0.9746	7.2992	0.0109*
Age(Yrs)	-0.0396	0.0666	-0.1718	0.0927	0.5538
Symptomless 0 Symptom 1	1.4446	0.9459	-0.4356	3.3248	0.1304
HT(Absent0Present1)	-0.0333	1.1761	-2.3709	2.3043	0.9775
HL(Absent0Present1)	0.6001	0.9456	-1.2793	2.4795	0.5273
DM(Absent0Present1)	2.2578	0.9363	0.3967	4.1189	0.0180*
smoke(Absent0Present1)	-0.2446	1.1123	-2.4554	1.9662	0.8265
T-CHO	-0.0202	0.0123	-0.0447	0.0043	0.1049
HbA1c	1.1860	0.6482	-0.1024	2.4744	0.0707
Angiostenosis rate NASCET (%)	-0.0032	0.0265	-0.0558	0.0494	0.9034
Echo luminance (hypo0 iso1 hyper2)	2.3520	0.7390	0.8821	3.8219	0.0021**

^*^P < 0.05; **P < 0.01

**Table 4:** Multiple regression analysis of plaque hardness and patient background

Variable	Partial regression coefficient	Standard error	95% confidence interval for partial regression coefficient		*P* value
			Lower limit	Upper limit	
GSM	-0.1233	0.0314	-0.1858	-0.0607	0.0002**
Pathological calcification (0–2)	-0.9154	0.6462	-2.2040	0.3731	0.1610
Sex	1.5869	1.6650	-1.7329	4.9067	0.3438
Age	-0.0814	0.0714	-0.2238	0.0610	0.2580
Symptom	-0.2213	0.9339	-2.0835	1.6408	0.8133
HT	-1.6622	1.1439	-3.9431	0.6186	0.1506
HL	0.2996	0.8945	-1.4841	2.0832	0.7387
DM	0.5196	1.2780	-2.0287	3.0678	0.6856
Smoking status	-0.6878	1.0263	-2.7342	1.3586	0.5049
T-CHO	-0.0160	0.0123	-0.0405	0.0085	0.1962
HbA1c	0.4164	0.8414	-1.2612	2.0941	0.6222
Angio stenosis rate NASCET (%)	0.0080	0.0259	-0.0436	0.0596	0.7585
Echo luminance (hypo0 iso1 hyper2)	1.5403	0.8500	-0.1545	3.2351	0.0742
Constant term	31.3886				

^**^P < 0.01

## Discussion

The pathogenic mechanism of cerebral infarction due to carotid artery stenosis includes hemodynamic infarction and embolism due to plaque rupture. For hemodynamic infarction, plaque stenosis rate is a clear indication for treatment; however, plaque properties are not standard indications for treatment. Soft plaques are prone causing stroke. We consider plaque hardness a standard indication for surgical treatment.

We quantitatively measured plaque hardness removed by CEA using a hardness meter and compared it with the pathological and patient background data and preoperative examination findings. Focusing on the blood vessel walls, collagen, which is a major component of plaque, and calcification as pathological factors, we quantified the hardness-determining factors of plaque. We focused on GSM as a quantification of collagen. GSM was examined to evaluate the diagnostic the echogenicity ratio using histogram analysis. Sermin et al. found that mean flexor tendons histogram analysis echogenicity/ mean median nerve histogram analysis echogenicity may useful quantitative parameters [25]. In addition, quantitative evaluation of plaque vulnerability by converting the echo brightness of the plaque into GSM has been conducted [26]. Plaque hardness quantified in this study and GSM were significantly correlated. Sex, diabetes, and echogenicity were extracted as significant findings in regression analysis performed with plaque hardness as the objective variable. GSM was the only significant factor in multiple regression analysis. Further, the hardness of the highly calcified sample was significantly higher than that of the uncalcified sample.

Our study showed that a higher echogenicity was associated with greater hardness of the plaque and that an increased amount of collagen was associated with a lower GSM. Several studies have assessed the pathological basis for the hardness of human carotid plaques. The basic pathological composition of the plaque is the fibrous cap and lipid core. The fibrous cap is mainly composed of smooth muscle cells, collagen, elastin, macrophages, foam cells, proteoglycans, and new vessels. The lipid core resides within the fibrous cap and consists of cholesterol, cell debris, foam cells, and calcium, and is severely deficient in proteoglycans and collagen [6, 27–29]. Collagen is a major constituent of the normal vessel wall and has a much higher stiffness than lipids, resulting in greater hardness [22, 23].

We considered collagen as a factor that determine the hardness of plaques and attempted to quantify it. Plaque collagen was EVG-stained and converted to a grayscale image, and the median value was determined by plotting the profiles in 256 steps. While some of the specimens measured were removed as whole pieces in a circumferential manner during surgery, others were removed piecemeal due to fragility. Therefore, the bounding rectangle of the plaque, including the intima, was set and measured to encompass the volume, stenosis rate, and properties of the plaque.

A stable plaque has a thick fibrous cap containing collagen, whereas an unstable plaque has a thin fibrous cap and is said to be a precursor lesion to rupture [30]. Studies on coronary arteries have also recognized unstable plaques as inflammatory lesions with thin fibrous caps that can cause acute coronary syndromes [31–34]. These findings suggest that the thickness of the fibrous cap in plaques is strongly related to their instability and tendency to rupture. Konishi et al. attempted to pathologically quantify plaque instability [35]. The authors reported that fibrous cap thickness, plaque rupture, microcalcification, and intra-plaque microvessels correlated with symptomatic plaques. Specifically, fibrous cap thickness less than 165 μm was found to be associated with unstable plaques [35]. Redgrave et al. also measured the thickness of the fibrous cap of plaques in symptomatic carotid stenosis and found that the fibrous cap in the ruptured group was thinner than that in the unruptured group. The authors also identified a cutoff value for the thickness of the fibrous cap of 200 μm [36]. In the present study, the quantitative plaque hardness was correlated with collagen content, which may indicate that the hard plaque has a thick fibrous cap with abundant collagen. In the multiple regression analysis in this study, plaque hardness was significantly correlated with GSM. Therefore, it can be said that the amount of collagen in the plaque strongly affects its hardness.

We also focused on calcification as a determinant of plaque hardness. Although pathological calcification was evaluated by HE staining, quantification of calcification was challenging in this study because it requires evaluation of the quantity of calcification and the quality of staining by shading. Therefore, the amount of calcification was evaluated by three semi-quantitative levels of "0,” "1,” and "2.” Consequently, there was a significant difference between "0,” indicating no calcification, and "2,” indicating some calcification.

Several studies have examined the relationship between plaque hardness and calcification; however, their results are controversial. For example, Mulvihill et al. used biochemical analysis by Fourier transform infrared (FTIR) spectroscopy and found that calcified specimens exhibited significantly higher stress [37]. Moreover, Barrett et al., measured composition and stiffness using FTIR analysis and micro X-ray computed tomography, which measures the calcification volume fraction relative to the total tissue content. The authors noted that the higher the lipid content, the lower the stiffness, with those with large calcification aggregates being the stiffest [38]. Conversely, a study comparing the hardness of carotid plaques using MRI and pathological findings found that calcification was not involved in plaque hardness [39]. Thus, calcification is not necessarily positively related to plaque hardness, and one reason for this may be the effect of the type of calcification. There are two types of plaque calcification: micro- and macro-calcification. Microcalcification is due to the progression of atherosclerosis and is the initial deposition of calcium in the necrotic core as a result of apoptosis of macrophages and vascular smooth muscle cells. In contrast, macrocalcification is a healing response to plaque inflammation involving grossly observable calcium deposition through the induction of osteoblast differentiation and vascular smooth muscle cell maturation. Microcalcification is more likely to be associated with plaque rupture, whereas macrocalcification is believed to lead to plaque stabilization and to be involved in plaque stiffness [40]. Macrocalcification is calcification that is visible to the naked eye, which in this semi-quantitative analysis is considered as "2.” Microcalcification is microscopic calcification and is considered as "1.” In other words, the difference in plaque hardness between "0″ and "2″ in this study indicates that macrocalcification affects plaque hardness.

In addition to GSM, sex, diabetes mellitus, and echoluminance showed significant differences in single regression analysis examining their correlation with plaque hardness. There were significant sex differences in carotid artery stenosis and most patients were male.

In the case of diabetes mellitus, there was no difference in HbA1c levels, although the presence or absence of the disease was significant, and treatment and underlying disease were not taken into account; these factors should be addressed in future studies. Our findings suggest that the echogenicity of carotid ultrasonography reflects pathological collagen content and plaque hardness.

This study also suggests that plaque hardness correlates with collagen and calcification levels. A method to preoperatively measure collagen, which shows the greatest correlation with plaque hardness, would be clinically valuable. In this study, echo findings correlated with plaque hardness during preoperative evaluation, although other preoperative evaluations such as CT and MRI were not performed. The development of a technique to quantitatively evaluate collagen and calcification based on imaging tests would be a useful preoperative evaluation for carotid artery stenosis in the future.

### Limitations

This study has several limitations, which should be considered. First, the amount of collagen and extent of calcification were quantified using slices of the narrowest part for which hardness was measured. Therefore, only a slice of each plaque is used to approximate the collagen and calcification of the whole plaque. This may lead to noise in acquiring the true quantity. Second, because this is a retrospective observational study, there is a bias in the cases studied. As the subjects in this study were those with an indication for surgery, only cases with a certain degree of severity were examined. Third, over 90% of specimens were derived from male patients in this study. This is because the prevalence of carotid artery stenosis is higher in men. In a cohort study of the general public aged 50–79 years, stenosis exceeding 50% was common in men [7.9%] and found in only 1.3% of women [41]. The cause for this difference may be the effects of smoking and underlying disease.

## Conclusion

Our study showed that a higher amount of collagen, which is the main component of the fibrous cap, was associated with greater plaque hardness and that increased hardness was associated with greater plaque stability. It was also shown that the amount of calcification may be related to plaque hardness. As part of preoperative evaluation, carotid echocardiography was considered a useful test because it correlates with plaque hardness.

## Data Availability

The datasets used and/or analyzed during the current study are available from the corresponding author on reasonable request.

## Abbreviations

CEA: Carotid endarterectomy
GSM: Gray-scale median
CAS: Carotid artery stenting
MPa: Megapascals
EVG: Elastica van Gieson staining
HE: Hematoxylin–eosin
FTIR: Fourier transform infrared
